# Metabolic dysfunction-associated steatotic liver disease and gastroesophageal reflux disease: a mendelian randomization study in European and East Asian populations

**DOI:** 10.3389/fgene.2024.1428334

**Published:** 2024-12-05

**Authors:** Chen’guang Su, Zheng Liao, Hewen Li, Yinxuan Pei, Zixiang Wang, Jian Li, Jinlong Liu

**Affiliations:** ^1^ Department of Hepatobiliary Surgery, Affiliated Hospital of Chengde Medical University, Chengde, Hebei, China; ^2^ Department of Minimally Invasive Spine Surgery, Affiliated Hospital of Chengde Medical University, Chengde, Hebei, China; ^3^ Hebei Key Laboratory of Panvascular Diseases, Chengde, Hebei, China

**Keywords:** metabolic dysfunction-associated steatotic liver disease, gastroesophageal reflux disease, mendelian randomization, causal effect, genome-wide association studies

## Abstract

**Background:**

Numerous observational studies have shown a potential association between metabolic dysfunction-associated steatotic liver disease (MASLD) and gastroesophageal reflux disease (GERD). However, causality is unclear. This study utilized genome-wide association study (GWAS) genetic data to explore the causal relationship between MASLD and GERD in European and East Asian populations.

**Methods:**

This study utilized a bidirectional, two-sample Mendelian randomization (MR) approach. All disease data were obtained from the GWAS database, and single nucleotide polymorphisms strongly associated with exposure were selected as instrumental variables. The inverse variance weighted (IVW) method is primarily utilized to evaluate the causal relationship between exposure and outcome. Finally, sensitivity analyses were performed to ensure the robustness of the results.

**Results:**

The IVW estimates indicated that non-alcoholic fatty liver disease (NAFLD) (odds ratio (OR) = 1.054, 95% confidence interval (CI), 0.966–1.150, *p* = 0.236) and percent liver fat (OR = 0.977, 95% CI, 0.937–1.018, *p* = 0.258) in European population were not linked to a higher risk of GERD. However, GERD in European population was associated with an increased risk of NAFLD (OR = 1.485, 95% CI, 1.274–1.729, *p* < 0.001) and percent liver fat (OR = 1.244, 95% CI, 1.171–1.321, *p* < 0.001). In addition, the IVW analysis in East Asian population showed that alanine aminotransferase (ALT) was associated with an increased risk of GERD (OR = 2.305, 95% CI, 1.241–4.281, *p* = 0.008), whereas aspartate aminotransferase (AST) had no causal effects on GERD risk (OR = 0.973, 95% CI, 0.541–1.749, *p* = 0.926). Furthermore, the associations between GERD and ALT (OR = 1.007, 95% CI, 0.998–1.015, *p* = 0.123) or AST (OR = 1.004, 95% CI, 0.997–1.012, *p* = 0.246) were not significant. After removing outliers, a significant correlation between GERD and ALT was observed (OR = 1.009, 95% CI, 1.001–1.016, *p* = 0.020).

**Conclusion:**

There was reverse causality between MASLD and GERD in European population, while there was bidirectional causality between a proxie for MASLD (ALT) and GERD in East Asian population. This study can provide novel insights into cross-ethnic genetic research on MASLD and GERD.

## 1 Introduction

Gastroesophageal reflux disease (GERD) is a prevalent disorder associated with gastrointestinal motility issues and can lead to complications such as reflux esophagitis (RE), esophageal stricture, Barrett’s esophagus, and esophageal adenocarcinoma (Fass, 2022; Mittal and Vaezi, 2020). Estimates suggest that GERD affects approximately 20% of adults in high-income countries (Maret-Ouda et al., 2020). Traditionally, significant risk factors for GERD include tobacco use (Kahrilas, 1992), obesity (Xie et al., 2024), and genetic predisposition (Ghoshal and Chourasia, 2011). However, preventive measures aimed at these risk factors have proven ineffective in controlling the progression of GERD (Sachar et al., 2024). Consequently, it has become essential to investigate new avenues regarding GERD risk factors. Recent epidemiological studies have indicated that metabolic diseases may also serve as risk factors for GERD (Chung et al., 2008; Wu et al., 2011).

Metabolic dysfunction-associated steatotic liver disease (MASLD), previously referred to as non-alcoholic fatty liver disease (NAFLD), is the most prevalent form of chronic liver disease globally, affecting approximately 25% of the population (Younossi et al., 2018). MASLD is a systemic metabolic disorder linked to obesity and insulin resistance (Maldonado-Rojas et al., 2024), characterized by excessive fat accumulation in liver cells (Younossi, 2019). The prognosis for MASLD is poor, as it can lead to liver fibrosis, cirrhosis, and ultimately progress to hepatocellular carcinoma (HCC) (Kanda et al., 2020; Cai et al., 2019). In recent years, numerous studies have explored the association between NAFLD and the risk of GERD. A Korean cohort study indicated a higher risk of reflux esophagitis in individuals with NAFLD compared to those without the condition (Min et al., 2018). However, this association is influenced by body mass index and other metabolic factors. Additionally, a meta-analysis that included nine observational studies and involved 185,118 participants demonstrated that NAFLD is linked to an increased risk of GERD (Xue et al., 2019). Nevertheless, the inherent limitations of traditional observational studies, such as reverse causality and confounding factors, obscure the causal relationship between MASLD and GERD. In addition, evidence underscores the genetic and environmental differences in the incidence and clinical manifestations of GERD. For example, a recent meta-analysis revealed that the prevalence of GERD is higher among Europeans (17.1%) than among Asians (10.0%) (Eusebi et al., 2018). Furthermore, Europeans with GERD may experience more severe heartburn (Spechler et al., 2002) and a higher incidence of Barrett’s esophagus and esophageal cancer compared to their Asian counterparts (Eusebi et al., 2021). Therefore, further investigation is necessary to clarify the discrepancies in the causal relationship between MASLD and GERD across different ethnic groups.

As a novel epidemiological method, Mendelian randomization (MR) leverages the random assignment and independence of genetic variants to determine causal associations between exposures and outcomes, effectively mitigating the influence of confounding factors (Davey Smith and Hemani, 2014). In addition, segregation of genetic variants occurs at conception, prior to the onset of disease, which helps to avoid reverse causation (Sanderson et al., 2022). Consequently, MR serves as a valuable alternative when conducting randomized controlled trials is constrained by ethical considerations and disease-specific characteristics. In this study, we employed a two-sample bidirectional MR analysis to evaluate the potential causal relationship between MASLD and GERD in European and East Asian populations.

## 2 Materials and methods

### 2.1 Ethical approval

All datasets used in this study were obtained from publicly available GWAS projects, each of which received the necessary ethical approval and consent. Therefore, no additional ethical approvals were required for our study.

### 2.2 Study design

**FIGURE 1:**
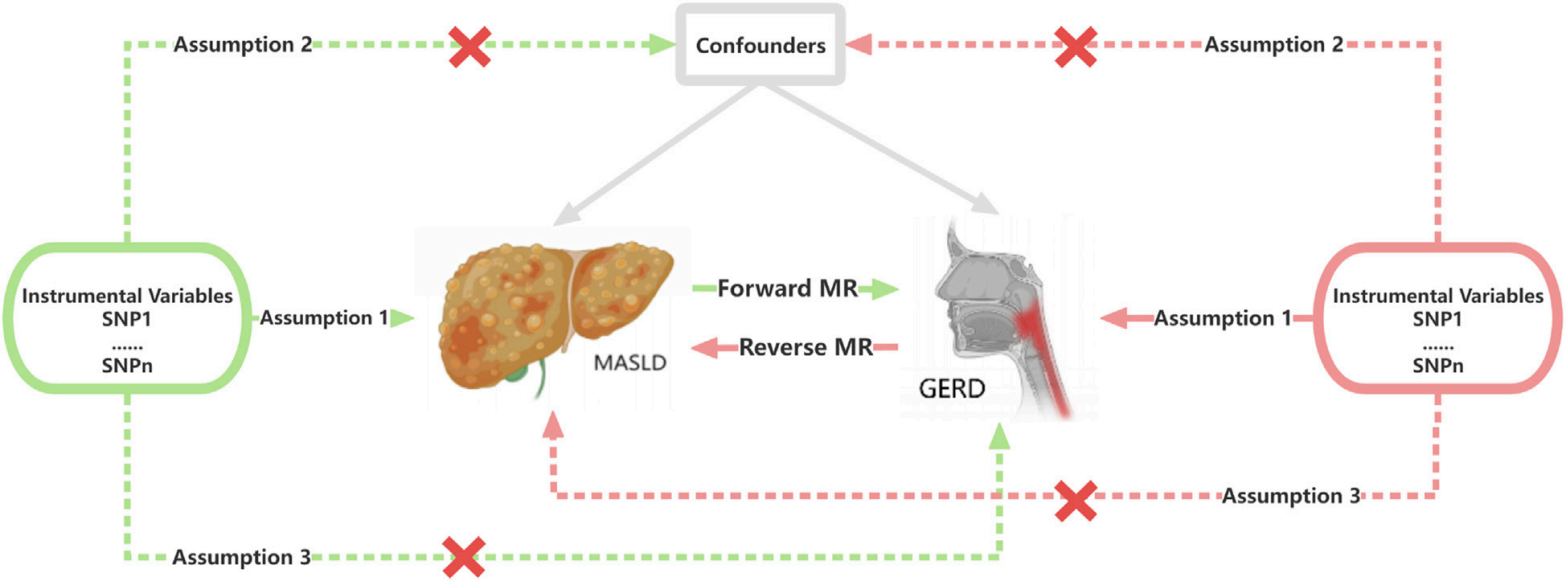
Flowchart of the bidirectional Mendelian randomization analysis in this study. SNP, single-nucleotide polymorphism; MASLD, Metabolic dysfunction-associated steatotic liver disease; GERD, gastroesophageal reflux disease; MR, Mendelian randomization.

We obtained data on genetic variants related to MASLD proxies and GERD in European and East Asian populations from various GWAS datasets, which we subsequently designated as exposure factors. The specific flow of a two-sample bidirectional MR analysis is depicted in Figure 1. To ensure the reliability of our results, we strictly adhered to the following three assumptions: (1) genetic variants must be strongly associated with the selected exposure factors; (2) genetic variants should not be associated with any confounding factors affecting both the exposure and the outcome; and (3) genetic variants must influence the outcome solely through the exposure pathway (Ference, 2015). This study followed the Strengthening the Reporting of Observational Studies in Epidemiology Using Mendelian Randomization (STROBE-MR) guidelines (Skrivankova et al., 2021).

### 2.3 Data source for European population

MASLD-related indicators include NAFLD and percent liver fat. Genetic associations with NAFLD were derived from the largest GWAS meta-analysis (Ghodsian et al., 2021), which included 8,434 NAFLD cases and 770,180 controls. This analysis utilized data from the Electronic Medical Records and Genomics (eMERGE) network, the United Kingdom Biobank, the Estonian Biobank, and FinnGen. In the eMERGE cohort, NAFLD was defined based on the use of electronic health record (EHR) ICD-9 and ICD-10 codes (ICD-9: 571.5, 571.8, and 571.9; ICD-10: K75.81, K76.0, and K76.9). The United Kingdom Biobank and Estonian Biobank employed ICD-10 codes [K74.0 (fibrosis), K74.2 (fibrosis), K75.8 (non-alcoholic steatohepatitis), K76.0 (NAFLD), and K76.9 (other specified diseases of the liver)]. In the FinnGen Consortium, NAFLD was defined solely by ICD-10 code K76.0. The GWAS data for genetic associations with percent liver fat were obtained from the United Kingdom Biobank, which also included 8,434 NAFLD cases and 770,180 controls (Liu et al., 2021). At the same time, data related to MASLD can be accessed through the Medical Research Council Integrative Epidemiology Unit (IEU) Open GWAS project (https://gwas.mrcieu.ac.uk/).

In addition, the largest publicly available GWAS statistics for GERD were obtained, which included 602,604 European participants from the EBI database (Ong et al., 2022). The diagnostic criteria for GERD are based on the 10th edition of the International Classification of Diseases.

### 2.4 Data source for East Asian population

Due to the lack of validated MASLD GWAS datasets in East Asian population, we opted for alanine aminotransferase (ALT) and aspartate aminotransferase (AST) levels from the Biobank Japan GWAS datasets as proxies for MASLD, based on previous research (Au Yeung et al., 2023; Zhao et al., 2024). Both enzymes have been established as diagnostic markers for MASLD (Schindhelm et al., 2006; Zou et al., 2020).

The data related to GERD in East Asian population were obtained from the EBI database, which included 948 cases and 177,516 controls (Sakaue et al., 2021).

The detailed information regarding the data sources used in this study is presented in Supplementary Table 1.

### 2.5 Genetic instruments selection and data harmonization

The IVs were subjected to a rigorous screening process in order to meet the three hypotheses previously outlined. As the number of IVs obtained at the significance threshold (*p* < 5.0 × 10^−8^) was insufficient for NAFLD in Europeans and GERD in East Asians, we adjusted the thresholds to *p* < 5.0 × 10^−6^ and *p* < 5.0 × 10^−5^, respectively, based on previous studies (Qin et al., 2023; Xiao et al., 2022). Subsequently, single nucleotide polymorphisms (SNPs) that were strongly associated with the exposure were clustered by setting the clumping parameter in the ‘TwoSampleMR’ package to TRUE. In addition, a strict *r* ^2^ threshold (*r* ^2^ < 0.001) was implemented to minimize the issue of multicollinearity caused by linkage disequilibrium and trimmed SNPs within a window size of 10,000 kb (Pritchard and Przeworski, 2001). Previously reported confounders such as smoking (Ge et al., 2024), obesity (Ding et al., 2023), and type 2 diabetes (Younossi et al., 2024) were screened, excluded by searching the PhenoScanner database (http://www.phenoscanner.medschl.cam.ac.uk/). SNPs that were not present in the outcomes, as well as SNPs with palindromic structures and incompatible alleles, were excluded during the process of harmonizing the exposure and outcome datasets. Subsequently, we screened for weak IVs by calculating the F-value, which was defined as F = *β* ^2^/*SE* ^2^ (Burgess et al., 2011), where *β* represents the estimated genetic effect on exposure and *SE* denotes the standard error. SNPs with an F-value calculated to be less than 10 were classified as weak IVs, suggesting insufficient strength to guarantee result precision, and were therefore excluded. Finally, we do not substitute did SNPs with a proxy to maintain the quality of the integrity process.

### 2.6 Statistical analysis

We employed inverse variance weighting (IVW) as the primary method for our initial analyses, supplemented by MR-Egger, weighted mode, simple mode, and weighted median approaches. Currently, IVW is the most widely utilized statistic in MR analysis. It is characterized by the exclusion of the intercept term during regression calculations, with the regression fitted using the reciprocal of the outcome variance as a weight. This approach serves to minimize the influence of bias from individual SNPs on the overall results (Hemani et al., 2018). Consequently, in the absence of horizontal pleiotropy, IVW provides the most accurate estimates in MR analysis. In contrast, MR-Egger is preferred when horizontal pleiotropy is significant, as it incorporates the intercept in the regression model (Bowden et al., 2016). Subsequently, we conducted a series of sensitivity analyses. Heterogeneity among genetic variants in the IVW estimates was assessed using Cochran’s Q statistic (Bowden et al., 2019). When the Q test (*p* < 0.05) indicated heterogeneity of results, random effects models were applied. Additionally, the outlier test of MR-Pleiotropy Residual Sum and Outlier (MR-PRESSO) was employed to identify and exclude outliers (*p* < 0.05 in the outlier test; NbDistribution = 10,000), and MR analysis were was repeated with adjusted analysis of the data (Patrick et al., 2021). If significant heterogeneity in Cochran’s Q test could not be eliminated after excluding outliers, the outlier test was performed again with a more stringent *p*-value threshold (*p* < 1 in the outlier test) (Chen et al., 2020). The MR-Egger intercept (Bowden et al., 2015) and the global test of MR-PRESSO (Verbanck et al., 2018) were utilized to identify and interpret potential horizontal pleiotropy. The presence of horizontal pleiotropy was demonstrated when the *p*-value for each of the two tests was less than 0.05. Finally, a leave-one-out analysis was applied to evaluate the impact of each SNP on the overall MR results. All statistical analyses were conducted using the ‘TwoSampleMR’ package (version 0.5.8) and the ‘MR-PRESSO’ package (version 1.0) in R software (version 4.2.3). The effect estimates of MR analysis were expressed as odds ratios (ORs) with 95% confidence intervals (CIs), and significant heterogeneity was defined as *p* < 0.05.

## 3 Results

### 3.1 Causal associations between MASLD and GERD in European population

Screening for MASLD and GERD-related indicators in European population, including NAFLD, percent liver fat, and GERD, was conducted following the screening process for genetic markers as outlined above. The F-statistics of all SNPs screened were greater than 10, indicating the absence of weak instrumental bias. A comprehensive list of all SNPs is provided in Supplementary Tables 2-5.

**FIGURE 2:**
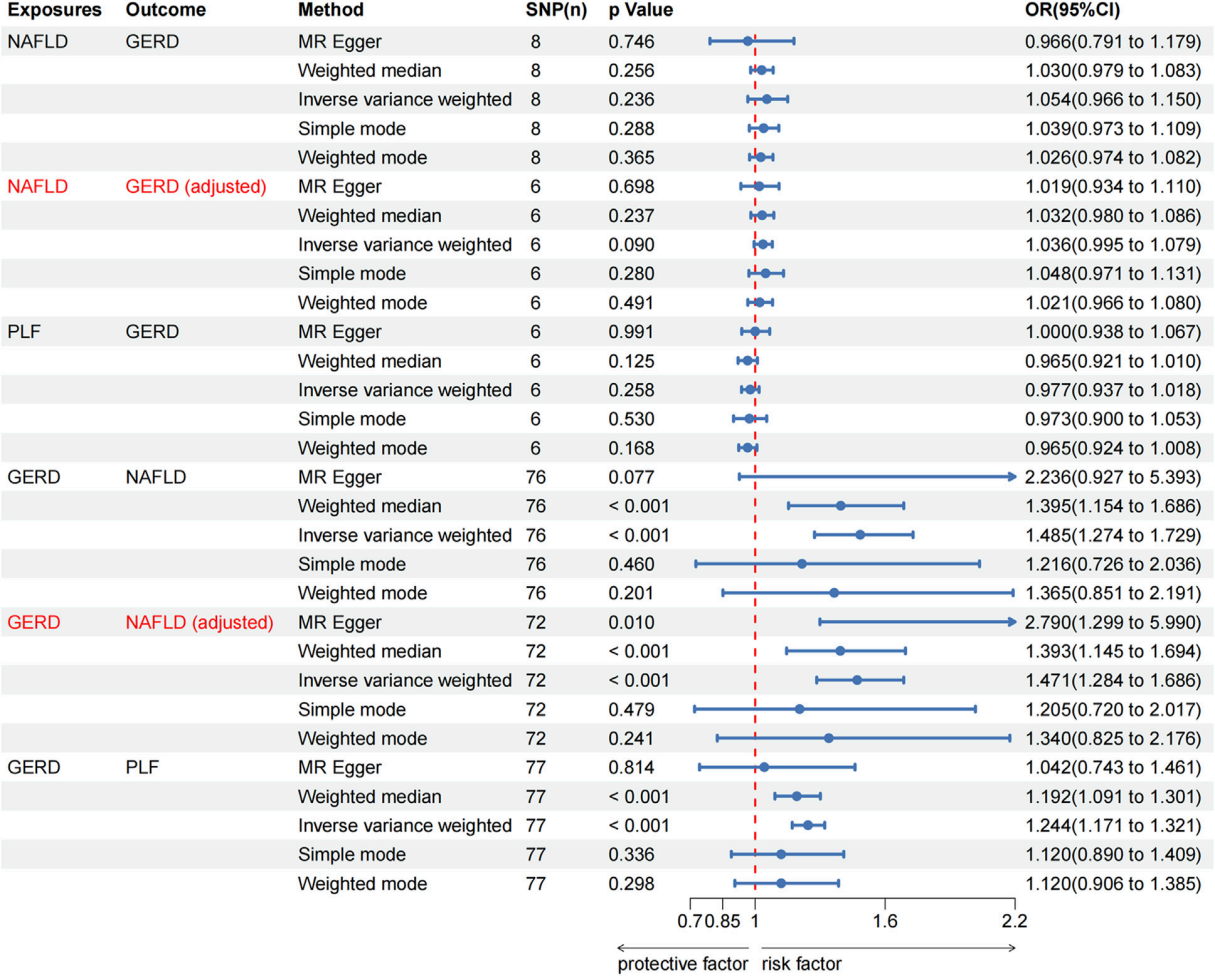
The Mendelian randomization analysis results of MASLD with GERD in European population. MASLD, Metabolic dysfunction-associated steatotic liver disease; GERD, gastroesophageal reflux disease; NAFLD, non-alcoholic fatty liver disease; PLF, percent liver fat; OR, odds ratio.

**FIGURE 3:**
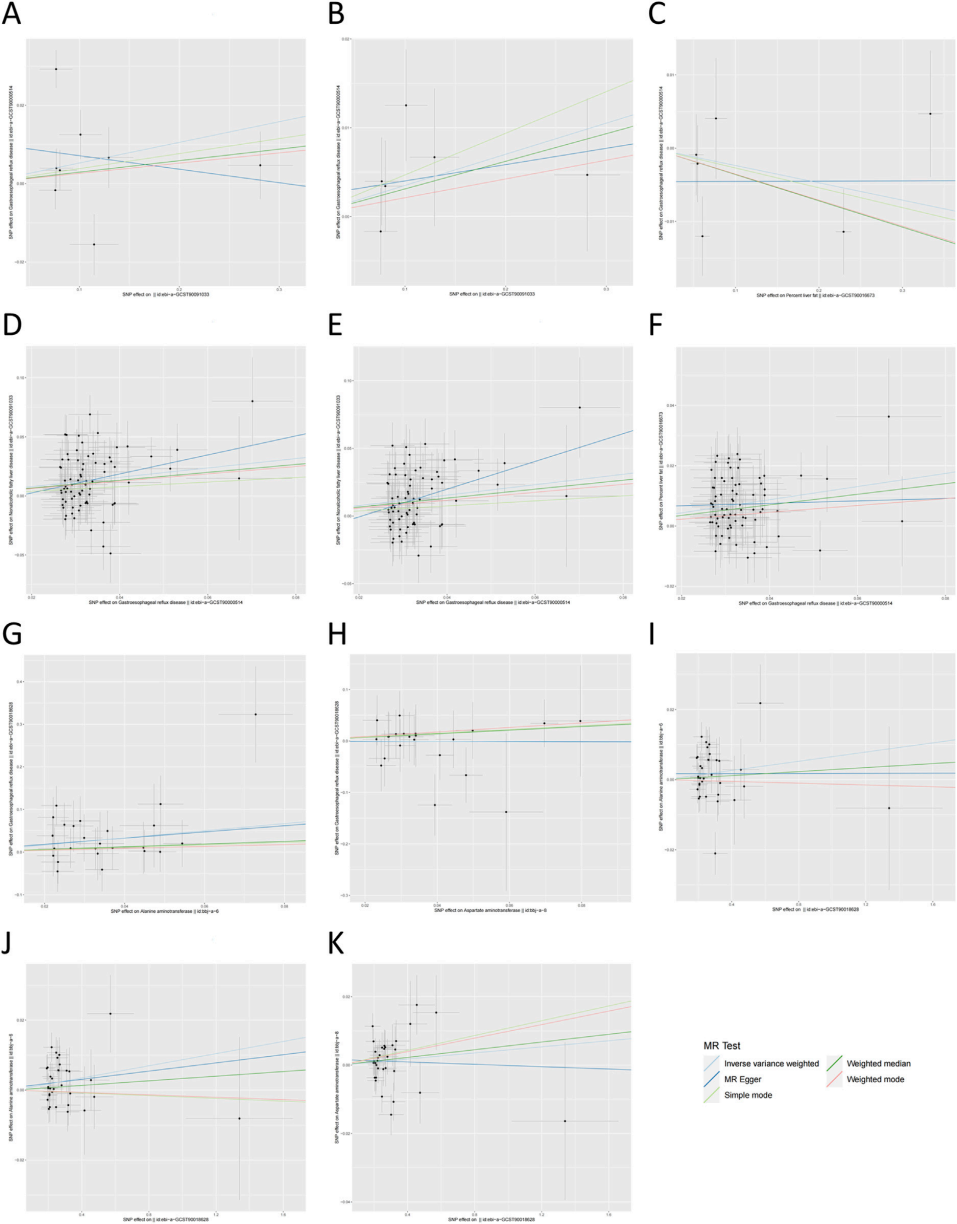
The scatter plot of Mendelian randomization analysis. **(A)** NAFLD on GERD; **(B)** NAFLD on GERD (adjusted); **(C)** percent liver fat on GERD; **(D)** GERD on NAFLD; **(E)** GERD on NAFLD (adjusted); **(F)** GERD on percent liver fat; **(G)** ALT on GERD; **(H)** AST on GERD; **(I)** GERD on ALT; **(J)** GERD on ALT (adjusted); **(K)** GERD on AST. NAFLD, non-alcoholic fatty liver disease; GERD, gastroesophageal reflux disease; ALT, alanine aminotransferase; AST, aspartate aminotransferase.

No significant causal association was observed between NAFLD and GERD according to IVW estimates (OR = 1.054, 95% CI, 0.966–1.150, *p* = 0.236). Moreover, no causal effect of percent liver fat on GERD was found (IVW: OR = 0.977, 95% CI, 0.937–1.018, *p* = 0.258). These findings were consistently supported by other supplementary methods. However, genetically predicted GERD was associated with an increased risk of NAFLD (IVW: OR = 1.485, 95% CI, 1.274–1.729, *p* < 0.001) and percent liver fat (IVW: OR = 1.244, 95% CI, 1.171–1.321, *p* < 0.001), and consistent results were obtained with weighted median (GERD on NAFLD: OR = 1.395, 95% CI, 1.154–1.686, *p* < 0.001; GERD on percent liver fat: OR = 1.192, 95% CI, 1.091–1.301, *p* < 0.001). All MR analysis results in European population were presented in Figures 2, 3A–F.

The results of the sensitivity analysis in European population are summarized in Table 1. The Cochran’s Q statistic using the IVW method revealed no observed heterogeneity, except for the MR analysis between NAFLD and GERD (NAFLD on GERD, *p* < 0.001; GERD on NAFLD, *p* = 0.002). Subsequently, we conducted the MR-PRESSO outlier test to address bias resulting from significant heterogeneity and then repeated the MR analyses (*p* < 0.05 in the outlier test). Specifically, heterogeneity disappeared after removing the outliers (rs7144175 and rs9922619) in the forward analysis (*p*_IVW_ = 0.645). In addition, since no outliers were detected in the reverse analysis, we no longer observed heterogeneity (*p*_IVW_ = 0.156) after excluding outliers with *p*-values less than 1 (rs2023878, rs569356, rs7685686, and rs9940128). Importantly, the exclusion of outliers did not change the results of the preliminary MR analyses between NAFLD and GERD [NAFLD on GERD, *p* = 0.090; GERD on NAFLD, *p* < 0.001; Figures 2, 3B,E] and was corrected for horizontal pleiotropy in the global test (NAFLD on GERD, *p* = 0.690; GERD on NAFLD, *p* = 0.170). Significant pleiotropy was not detected in European population, as indicated by the Egger intercept (all *p* > 0.05). Furthermore, when the leave-one-out method was applied to remove any single SNP, none of the IVW effect values were affected (Supplementary Figure 1A-F). The forest plot and funnel plot are shown in Supplementary Figures 2A-F, 3A-F.

**TABLE 1:** Results of sensitivity analysis in Mendelian randomization analysis.

Exposure	Outcome	Ancestor	Method	nSNP	Cochran’s Q test			Intercept term			Global test	
					Q	Q_df	*p*	Intercept	SE	*p*	RSSobs	*p*
NAFLD	GERD	European	MR Egger	8	33.706	6	7.67E-06	0.011	0.011	0.377		
			IVW	8	38.817	7	2.12E-06					
			MR-PRESSO	8							48.486	<0.001
NAFLD	GERD (adjusted)	European	MR Egger	6	3.173	4	0.529	0.002	0.005	0.689		
			IVW	6	3.358	5	0.645					
			MR-PRESSO	6							4.992	0.690
Percent liver fat	GERD	European	MR Egger	6	6.174	4	0.186	−0.005	0.005	0.399		
			IVW	6	7.547	5	0.183					
			MR-PRESSO	6							14.770	0.337
GERD	NAFLD	European	MR Egger	76	114.444	74	0.002	−0.014	0.015	0.357		
			IVW	76	115.772	75	0.002					
			MR-PRESSO	76							118.842	0.001
GERD	NAFLD (adjusted)	European	MR Egger	72	79.812	70	0.198	−0.021	0.013	0.100		
			IVW	72	82.980	71	0.156					
			MR-PRESSO	72							85.289	0.170
GERD	Percent liver fat	European	MR Egger	38	81.940	75	0.273	0.006	0.006	0.299		
			IVW	38	83.132	76	0.269					
			MR-PRESSO	38							85.376	0.315
ALT	GERD	East Asian	MR Egger	25	21.554	23	0.547	0.004	0.034	0.909		
			IVW	25	21.568	24	0.605					
			MR-PRESSO	25							23.326	0.614
AST	GERD	East Asian	MR Egger	22	11.327	20	0.937	−0.001	0.033	0.977		
			IVW	22	11.328	21	0.956					
			MR-PRESSO	22							12.578	0.953
GERD	ALT	East Asian	MR Egger	34	54.175	29	0.008	0.002	0.004	0.674		
			IVW	34	54.480	30	0.011					
			MR-PRESSO	34							57.828	0.013
GERD	ALT (adjusted)	East Asian	MR Egger	33	39.567	31	0.139	0.001	0.003	0.834		
			IVW	33	39.624	32	0.166					
			MR-PRESSO	33							42.202	0.187
GERD	AST	East Asian	MR Egger	34	45.051	32	0.063	0.002	0.004	0.656		
				IVW	34	45.335	33	0.075					
			MR-PRESSO	34							48.131	0.056

SNPs, single nucleotide polymorphisms; SE, standard error; p, *p*-value; IVW, inverse variance weighted; PRESSO, Pleiotropy RESidual Sum and Outlier; NAFLD, non-alcoholic fatty liver disease; GERD, gastroesophageal reflux disease; ALT, alanine aminotransferase; AST, aspartate aminotransferase.

### 3.2 Causal associations between MASLD and GERD in East Asian population

We effectively screened for genetic variants associated with MASLD and GERD, including ALT, AST, and GERD in East Asian population through the process described above. All F-statistics exceeded the threshold of 10, indicating the absence of weak instrumental bias. The detailed information for all SNPs is available in Supplementary Tables 6-9.

**FIGURE 4:**
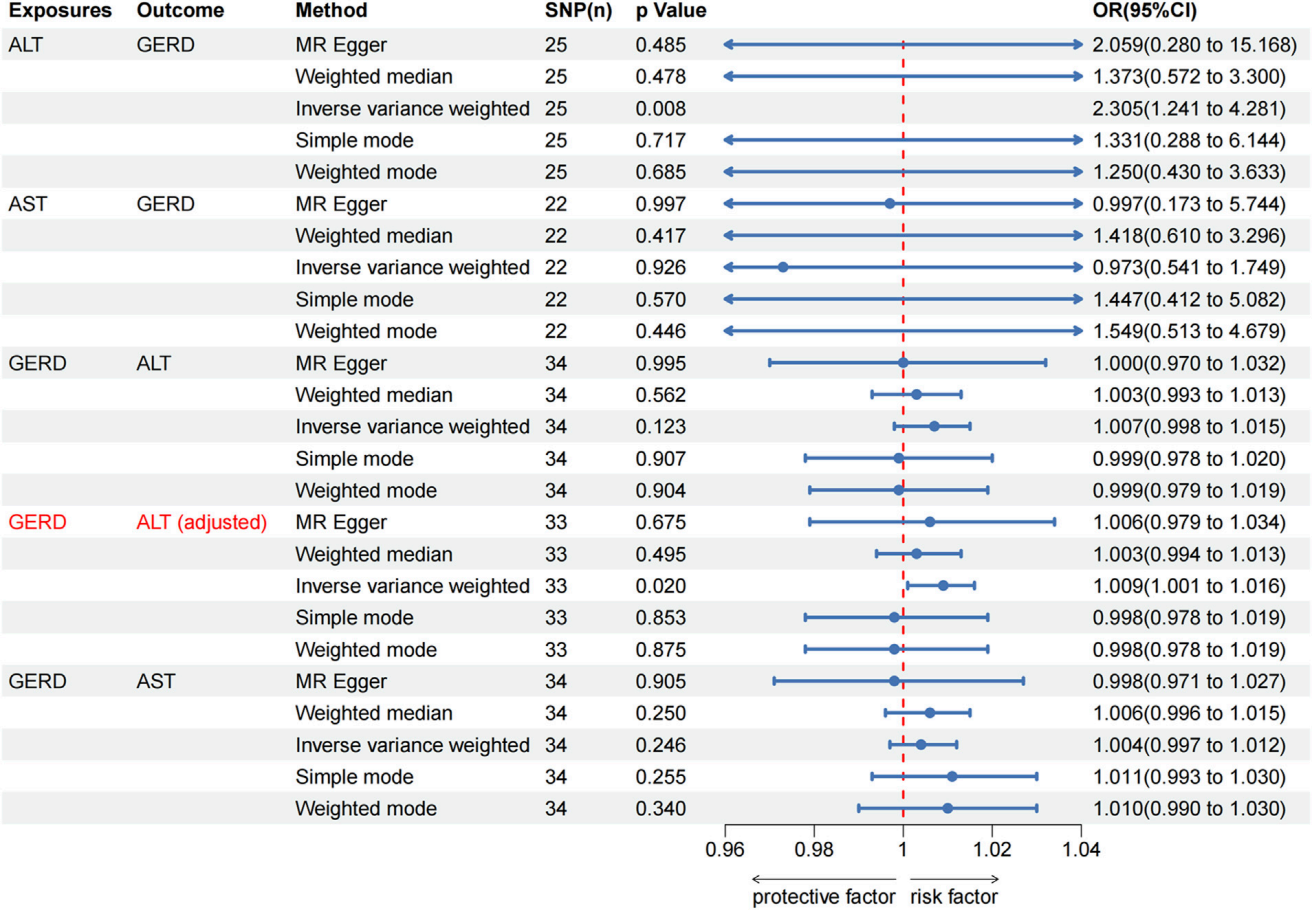
The Mendelian randomization analysis results of MASLD with GERD in East Asian population. MASLD, Metabolic dysfunction-associated steatotic liver disease; GERD, gastroesophageal reflux disease; ALT, alanine aminotransferase; AST, aspartate aminotransferase; OR, odds ratio.

As shown in Figure 4, the IVW analysis indicated that genetically predicted ALT was positively associated with GERD (OR = 2.305, 95% CI, 1.241–4.281, *p* = 0.008) in East Asian population. Surprisingly, the IVW analysis indicated no evidence of a causal link between AST and the risk of GERD (OR = 0.973, 95% CI, 0.541–1.749, *p* = 0.926) in East Asian population. Furthermore, no evidence of a causal link between GERD and the risk of ALT (IVW: OR = 1.007, 95% CI, 0.998–1.015, *p* = 0.123) and AST (IVW: OR = 1.004, 95% CI, 0.997–1.012, *p* = 0.246) in East Asian population was observed during this study. These findings were consistently supported by other supplementary methods. Detailed information on all MR analysis in East Asian population is presented in Figures 3G–K.

We conducted a sensitivity analysis to assess the robustness of the MR results in East Asian population (Table 1). The Cochran’s Q test revealed the presence of heterogeneity in the causal relationship between GERD and ALT in East Asian population (*p*_IVW_ = 0.011). Subsequently, we identified outliers through the MR-PRESSO outlier test (rs6022197). Heterogeneity was no longer detected when outliers were eliminated (*p*_IVW_ = 0.166), and horizontal pleiotropy in the global test was also eliminated (*p* = 0.187). Interestingly, following the adjustment, GERD was associated with high levels of ALT (IVW: OR = 1.009, 95% CI, 1.001–1.016, *p* = 0.020; Figures 4, 3J). Conversely, Cochran’s Q statistic showed no significant heterogeneity in the remaining MR analyses in East Asian population (all *p* > 0.05). In addition, the MR-Egger intercept did not provide evidence of horizontal pleiotropy in any of the MR analyses (all *p* > 0.05). Finally, the robustness of the MR results is further emphasized by the leave-one-out sensitivity analysis (Supplementary Figure 1G-K). The forest plot and funnel plot are shown in Supplementary Figures 2G-K, 3G-K.

## 4 Discussion

This study is the first to establish a causal relationship between MASLD and GERD through large-scale MR analysis. Specifically, for European population, our results indicated that factors related to MASLD had no effect on the risk of developing GERD. However, GERD was associated with an increased risk of MASLD. Furthermore, for East Asian population, ALT, a surrogate marker for MASLD, was positively associated with GERD; however, there was no evidence of a causal relationship between GERD and altered ALT and AST levels. In addition, sensitivity analyses demonstrate the robustness of our findings.

MASLD has been characterized as a systemic metabolic disease closely associated with metabolic disorders and insulin resistance (Al Hashmi et al., 2024). In 2020, a panel of international experts from 22 countries renamed NAFLD to MASLD through a Delphi process, as NAFLD does not accurately reflect the etiology of the disease. The new definition incorporates cardiometabolic criteria and underscores the importance of obesity, diabetes, and Metabolic Syndrome (MetS) in the development of MASLD (Eslam et al., 2020). Numerous observational studies have confirmed that MASLD is a risk factor for GERD. A meta-analysis conducted by Xue et al., which included 185,118 subjects, demonstrated a significant association between NAFLD and an increased risk of GERD, although the findings may have been influenced by severe publication bias (Xue et al., 2019). Furthermore, a large cohort study of Korean adults revealed a higher incidence of reflux esophagitis among participants with NAFLD, even after adjusting for age and sex. Notably, the correlation between the two conditions diminished when the researchers further adjusted for confounding factors, including smoking status, alcohol consumption, regular exercise, education level, and body mass index (BMI) (Yang et al., 2017). Additionally, Qiu et al. found that NAFLD significantly elevated the risk of RE by strictly controlling for the subjects’ BMI. Interestingly, the study also indicated a higher prevalence of NAFLD in patients with RE compared to those without RE, and this difference was statistically significant (Qiu et al., 2023).

It is noteworthy that a significant number of studies have focused on East Asian population. Our research indicates a positive correlation between the proxy for MASLD (ALT) and GERD in this demographic. Unfortunately, due to the unavailability of a MASLD dataset specifically for East Asian populations, we were constrained to seek proxy data that exhibited a high degree of correlation with MASLD for the purpose of our analysis. It is important to acknowledge that this practice is still limited by several potential risks. First, transaminases do not fully capture the complex pathomechanisms of MASLD, which include alterations such as dysregulation of fatty acid metabolism and impaired bile acid cycling (Feng et al., 2024). Additionally, the proxy status of transaminases may be more relevant to individuals with metabolic abnormalities, potentially leading to bias in our conclusions (Clark et al., 2003). Furthermore, it cannot be ruled out that patients with MASLD may present with normal aminotransferase levels, raising concerns about the potential overestimation of aminotransferases as proxy indicators. Nevertheless, there is substantial evidence supporting the feasibility of using transaminases as proxies for MASLD. A significant body of research demonstrated a strong correlation between transaminase levels and MASLD risk factors, including obesity, dyslipidemia, and elevated insulin levels (Lin et al., 2022; Ma et al., 2019). Additionally, a large-scale epidemiological study indicated that ALT and AST could serve as accurate predictors of MASLD prevalence when common chronic liver disease conditions are excluded (Clark et al., 2003). However, the development of a comprehensive MASLD dataset specifically for East Asian populations will be essential for enhancing our understanding of the current findings and for improving future research.

In clinical practice, the risk factors for GERD are often numerous and can be modified (Sadafi et al., 2024). However, the systemic metabolic characteristics of MASLD have obscured the potential mechanisms by which it affects a single system. Currently, advances in the study of new biological mechanisms may provide insights into the link between MASLD and GERD in East Asian populations. First, MASLD leads to the activation of pro-inflammatory mediators, including IL-1β, IL-6, and hydrogen peroxide, which in turn triggers neurogenic esophageal muscle contractions, resulting in frequent dilation of the esophagus to the point of relaxation (Cao et al., 2004). Secondly, leptin may serve as another link connecting MASLD to GERD. MASLD is associated with elevated leptin levels (Jiménez-Cortegana et al., 2021), and increased leptin can disrupt the acidic environment of the esophageal lumen, leading to the initiation and progression of esophageal mucosal damage (Pardak et al., 2021). Finally, MASLD activates systemic oxidative stress levels (Bae et al., 2023), which diminishes the esophagus’s ability to repair damage to its mucosa (Han and Zhang, 2022).

It is important to note that studies elucidating the causal relationship between MASLD and GERD are not confined to East Asia. Observational studies conducted in Europe have also documented this association. A cross-sectional study by an Italian internal medicine team thoroughly analyzed the prevalence and clinical characteristics of GERD symptoms in individuals with NAFLD. The findings indicated that the prevalence of gastroesophageal reflux symptoms was significantly higher in the NAFLD group compared to the non-NAFLD group (Miele et al., 2012). Furthermore, another cross-sectional study by Catanzaro et al. reported that the prevalence of GERD symptoms was significantly greater in NAFLD patients than in the control group (Catanzaro et al., 2014). However, these findings contradict those observed in European population in this study, where no significant association was found between MASLD and GERD risk. Interestingly, reverse MR analysis suggested a causal relationship between GERD and MASLD. Potential factors contributing to this discrepancy may include several scenarios. Firstly, observational studies, particularly cross-sectional studies, can only demonstrate associations. Their ability to investigate causality is limited, and they cannot completely eliminate subtle biases introduced by confounding factors, even if some covariates are adjusted for in the study. Second, the association between the two diseases in European population observed in the study may be due to reverse causation, as suggested by the results of our MR. The potential relationship between GERD and MASLD in European population should be further validated in future research, including an examination of clinical features and biological mechanisms. Third, the association between MASLD and GERD in European populations may be mediated by specific metabolic pathways. Evidence suggests that MASLD is strongly linked to insulin resistance (IR) within these populations. In particular, hepatic steatosis can impair insulin action in the liver (Barber et al., 2023). Additionally, a pilot study involving European and African American obese women indicated that insulin resistance contributes to both the prevalence and severity of GERD in this demographic (Pointer et al., 2016). However, the precise mechanisms through which IR serves as an intermediary in this relationship remain unclear.

It is noteworthy that our study found different results between European and East Asian populations. In particular, we observed a unidirectional association between MASLD and GERD in European population. In contrast, the East Asian population showed bidirectional causality, and this discrepancy requires further discussion. A potential factor contributing to these inconsistent results could be the use of MASLD proxies among East Asians. Although we demonstrated the reliability of ALT and AST as proxies, the results should still be interpreted with caution. On the other hand, genetic differences between races can also play an important role. Several studies have shown that Caucasians with a genetic predisposition to MASLD predominantly express the PNPLA3, GCKR, and PPP1R3B genes (Oddy et al., 2013), while East Asians are characterized primarily by polymorphisms in the APOC3 gene (Hsu and Kao, 2012). Regarding metabolic predisposition, the metabolic mechanisms of MASLD in Caucasians are primarily influenced by IR, whereas in East Asians, they are more closely associated with BMI levels (Al Rifai et al., 2015). In addition, variations in dietary habits, including the amount of alcohol consumed, high-fat diets, and coffee intake, may also play a significant role (Fan and Cao, 2013; Festi et al., 2009). It is therefore imperative that further investigation be conducted into the underlying causes and mechanisms of these racial disparities.

Our study has several strengths that merit discussion. First, we employed a rigorous design concept of MR analysis, which enhances the authority and credibility of our exploration into the causal association between MASLD and GERD. Second, our study utilized bidirectional MR analysis, allowing for a comprehensive examination of the directionality of the causal relationship between these diseases and assessing the potential for reverse causation. Third, the data sources for all diseases included multiple traits rather than a single trait, enriching the breadth of our findings. Finally, this study investigated differences in causal associations across racial groups, aiming to address the oversight of racial disparities in disease prevalence and outcomes.

However, it is important to acknowledge some inherent limitations in our study. First, due to the lack of MASLD datasets in East Asian population, we substituted MASLD with liver enzyme levels. Although ALT and AST are well-established markers of MASLD, this substitution was based on similar previous studies. Furthermore, liver enzymes still lack absolute specificity for MASLD; therefore, caution must be exercised when interpreting the results. Second, publicly available GWAS meta-analyses lacked specific information on age and gender, which hindered our ability to stratify the results using appropriate subgroup analyses. Finally, MR analyses can only provide linear assessments of associations between diseases and do not elucidate the specific molecular mechanisms underlying the association between MASLD and GERD.

In summary, our study provided novel clinical insights into the cross-racial association between GERD and MASLD. Based on these findings, clinicians should pay closer attention to the management of GERD when evaluating European patients to prevent and mitigate the progression of MASLD. Furthermore, for the clinical management of East Asian patients, we advocate for a more integrated treatment approach that considers both GERD and MASLD. Meanwhile, the specific mechanisms underlying this bidirectional relationship warrant further investigation to optimize treatment strategies for East Asian patients.

## 5 Conclusion

In conclusion, our evidence supports a reverse causality relationship between MASLD and GERD in European population. However, our study provides novel evidence suggesting a bidirectional causality between a proxie for MASLD (ALT) and GERD in East Asian population. Future research should include larger prospective studies and basic research to further elucidate the underlying mechanisms of the association between MASLD and GERD.

## Data Availability

The original contributions presented in the study are included in the article/Supplementary Material, further inquiries can be directed to the corresponding author.

## Supplementary material

SUPPLEMENTARY FIGURE 1 Leave-one-out sensitivity analysis of Mendelian randomization analysis. **(A)** NAFLD on GERD; **(B)** NAFLD on GERD (adjusted); **(C)** percent liver fat on GERD; **(D)** GERD on NAFLD; **(E)** GERD on NAFLD (adjusted); **(F)** GERD on percent liver fat; **(G)** ALT on GERD; **(H)** AST on GERD; **(I)** GERD on ALT; **(J)** GERD on ALT (adjusted); **(K)** GERD on AST. NAFLD, non-alcoholic fatty liver disease; GERD, gastroesophageal reflux disease; ALT, alanine aminotransferase; AST, aspartate aminotransferase.

Supplementary Figure 2 The forest plot of Mendelian randomization analysis. **(A)** NAFLD on GERD; **(B)** NAFLD on GERD (adjusted); **(C)** percent liver fat on GERD; **(D)** GERD on NAFLD; **(E)** GERD on NAFLD (adjusted); **(F)** GERD on percent liver fat; **(G)** ALT on GERD; **(H)** AST on GERD; **(I)** GERD on ALT; **(J)** GERD on ALT (adjusted); **(K)** GERD on AST. NAFLD, non-alcoholic fatty liver disease; GERD, gastroesophageal reflux disease; ALT, alanine aminotransferase; AST, aspartate aminotransferase.

Supplementary Figure 3 The funnel plot of Mendelian randomization analysis. **(A)** NAFLD on GERD; **(B)** NAFLD on GERD (adjusted); **(C)** percent liver fat on GERD; **(D)** GERD on NAFLD; **(E)** GERD on NAFLD (adjusted); **(F)** GERD on percent liver fat; **(G)** ALT on GERD; **(H)** AST on GERD; **(I)** GERD on ALT; **(J)** GERD on ALT (adjusted); **(K)** GERD on AST. NAFLD, non-alcoholic fatty liver disease; GERD, gastroesophageal reflux disease; ALT, alanine aminotransferase; AST, aspartate aminotransferase.
