# Supplementary figures and images for: Metabolic dysfunction-associated steatotic liver disease and gastroesophageal reflux disease: a mendelian randomization study in European and East Asian populations
Source: Front Genet. 2024 Dec 5;15:1428334. doi: 10.3389/fgene.2024.1428334 (PMC11655479; doi:10.3389/fgene.2024.1428334)

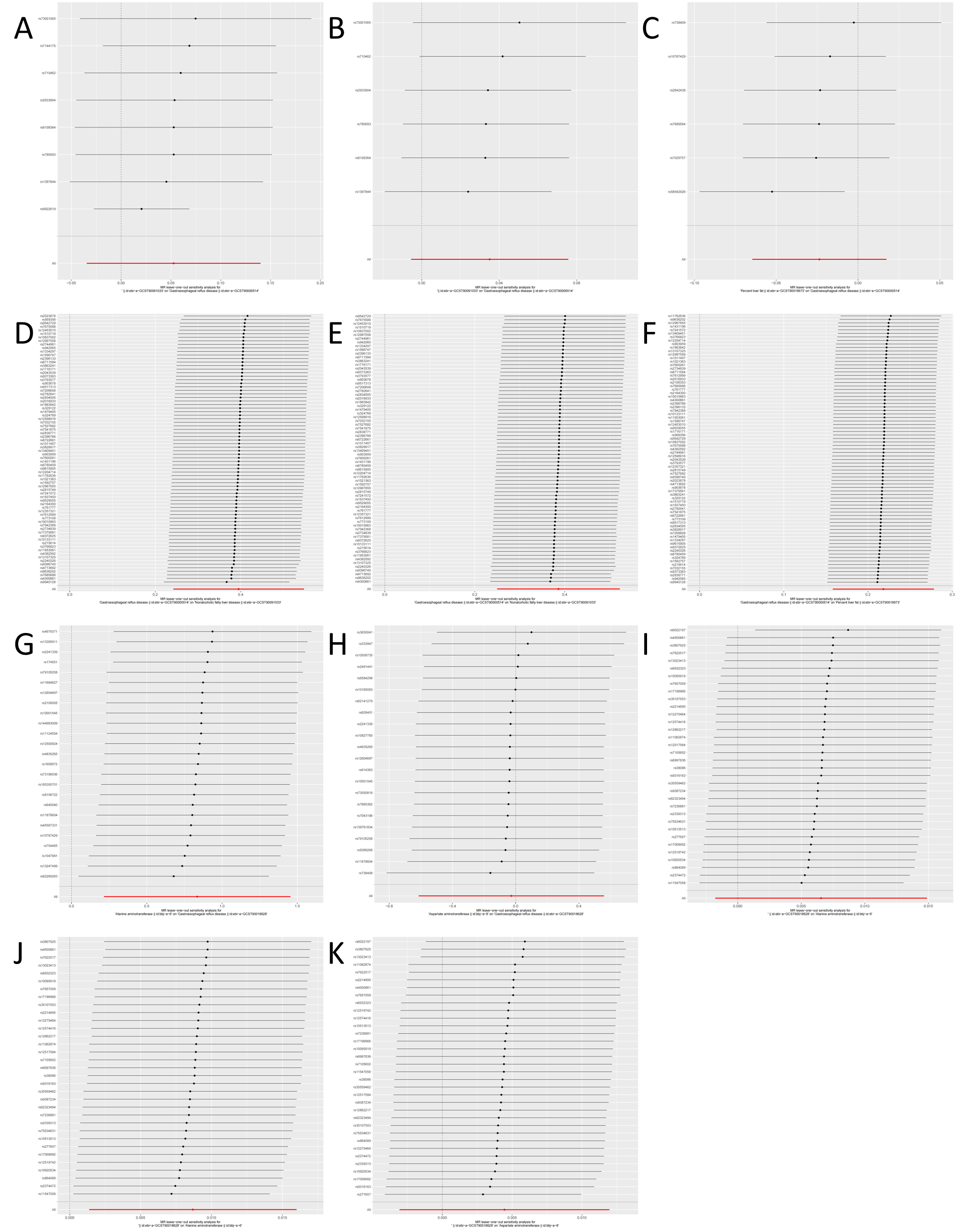

Supplement: Supplementary file 1 [file DataSheet2.zip › Supplementary Figure/Supplementary Figure 1.tif]

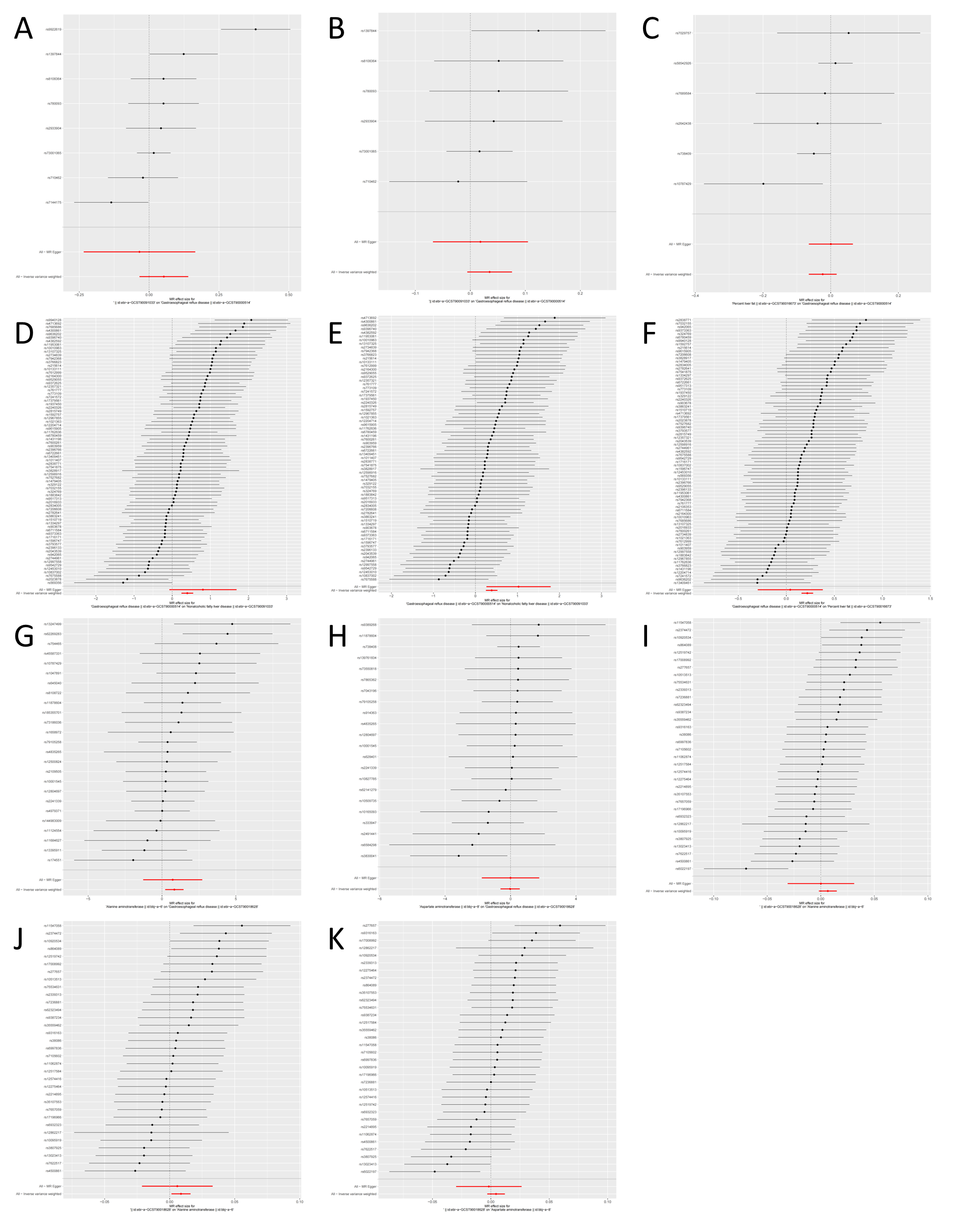

Supplement: Supplementary file 1 [file DataSheet2.zip › Supplementary Figure/Supplementary Figure 2.tif]

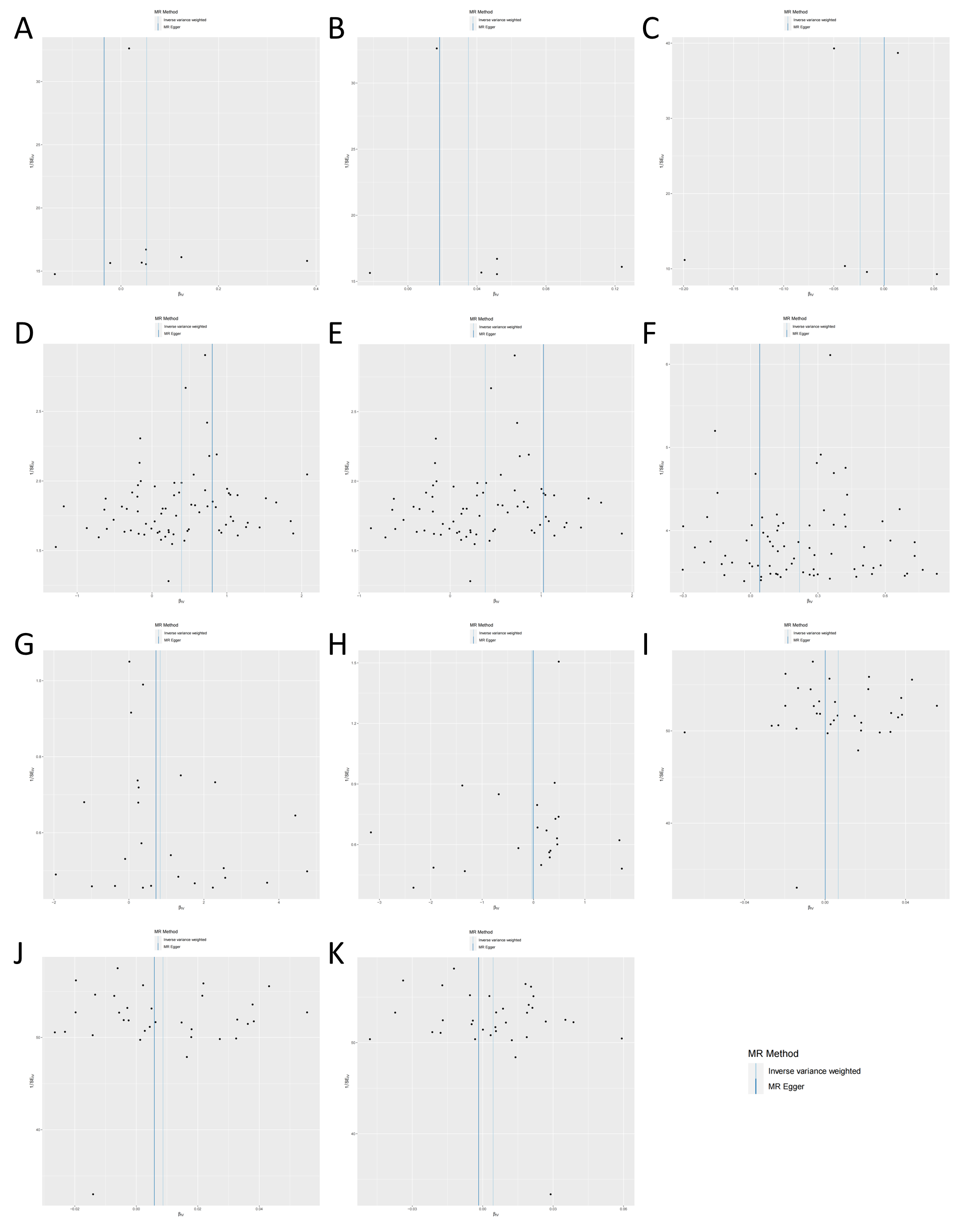

Supplement: Supplementary file 1 [file DataSheet2.zip › Supplementary Figure/Supplementary Figure 3.tif]
